# Could surgery be the gold standard in moderate and severe ischaemic colitis? Atypical case description and review of literature

**DOI:** 10.1007/s00384-014-1877-z

**Published:** 2014-05-14

**Authors:** F. Virdis, E. Mekonnen, R. D’Souza, S. Tacci, M. Varcada

**Affiliations:** Department of Emergency General Surgery, Royal Free Hospital, Pond Street, London, Greater London, NW3 2QG UK

Dear Editor,

Ischaemic colitis (IC) is a rare condition caused by an inadequate perfusion leading to colonic inflammation. This condition has an incidence of 4.5–9.9 per 100,000 population/year [1]. The exact pathophysiology is still debated; the main mechanism implicated is an ischaemia-reperfusion phenomenon with rapid return of normal mesenteric blood flow [2]. Despite suggestive evidence for a vascular or autonomic cause for IC, most cases have no identifiable cause. These spontaneous episodes are thought to be the result of impairment of the micro-vascularization of the colonic wall. Several risk factors have been described, which include comorbidities and medications. Two types of IC are described, defined by their severity: sever IC with transmural colonic ischaemia and/or multi-organ failure (MOF) and mild-moderate IC without MOF [2]. The clinical presentation is variable, and clinical signs are nonspecific [2]. Lower gastrointestinal bleeding is often accompanied by urgent diarrhoea. However, blood loss is usually minimal without haemodynamic compromise or the need for transfusions and profuse bleeding should suggest another diagnosis [3].

Surgical treatment is necessary in about 20–28 % of patients with IC [3]. There is no overall consensus regarding treatment of IC [2]. Diaz-Nieto et al. reported that surgery is only generally agreed upon in peritonitis and conservative treatment is used for remaining cases, with an undefined role for surgery in the latter [1].

A 70-year-old male was admitted to our institution with sudden sided left abdominal pain associated with two episodes of bloody diarrhoea, nausea, belching, vomiting, dysuria and pyrexia. The patient was on Simvastatin on a regular basis only. On admission, all the patient’s observations were stable, with blood pressure 153/64 mmHg, heart rate 70 beats/min, body temperature 36.5 °C and oxygen saturation 100 % in room air. On clinical examination, the abdomen was soft and quite tender in the left lower quadrant. There were no palpable masses or signs of peritoneal irritation. Normal bowel sounds. On rectal (PR) examination, there was minimal bright red blood on the glove, with an empty rectum. Blood chemistry revealed a high white blood cell count (16.47 × 10^9^/L) with neutrophils 14.07 × 10^9^/L, haemoglobin 156 g/L, C-reactive protein level 2 mg/L, lactate 2.47, BE 1.6, normal PH and normal clotting. Initially, management was based on bowel rest, intra venous (IV) fluids, IV antibiotics (ciprofloxacin and metronidazole) and analgesia (paracetamol, tramadol). An abdominal computer tomography (CT) scan with contrast, performed the day after admission, showed a significant abnormality of the colon from the ascending colon in a continuous fashion to the descending/sigmoid junction. The caecum, sigmoid and rectum appeared unremarkable. The colon was thick walled and oedematous with some fluid in the left paracolic gutter and within the pelvis. No collections. No pneumatosis. The colon affected appeared underperfused, and the final radiologist’s report concluded acute colitis. Therefore, *Clostridium difficile* infection was excluded with blood investigations. A sigmoidoscopy performed 4 days after the admission showed severely inflamed proximal sigmoid and distal descending colon with significantly hyperaemic mucosa, and the histological report of biopsies taken during the procedure indicated that the distal colon and rectum were within normal limits; the sigmoid colon was affected by ulceration and features in keeping with the clinical suspicion of IC. During the first week of treatment, our patient improved in terms of abdominal pain, associated with no further PR bleeding, bowels regularly opened and observations stable. His blood investigations improved as WCC declined (down to 9.3 × 10^9^/L), but CRP increased to 247 mg/L. During the second week following the admission, therapeutic tinzaparin was started. However, despite medical treatment and apparent improvements, on the 12th hospitalization day, the patient presented a few episodes of PR bleeding. An abdominal CT angiogram was performed, and it showed active bleeding from the anterior wall of the transverse colon, arising from a branch of the middle colic artery. Embolisation using micro coils was then successfully performed. Moreover, on the 14th day of hospitalization, the general condition of the patient started to deteriorate, with increasing abdominal pain associated with diffuse tenderness, pyrexia and tachycardia. Therefore, after 14 days of conservative management, surgical intervention with laparotomy, total colectomy and end ileostomy was performed. On-table colonoscopy was performed and showed pancolitis.

There is no overall consensus regarding treatment of IC; no studies with a sufficient level of evidence are available in the literature to establish clinical practice guidelines [1]. Any part of the bowel may be affected, but the splenic flexure and descending and sigmoid colon are the most common sites. In our case, the initial CT scan showed that the disease diffusion was quite uncommon, with sparing of the sigmoid colon. However, the biopsy performed during the sigmoidoscopy, which was done 2 days after the CT scan, reported an involvement of the proximal sigmoid.

Following an initial improvement with conservative management (intravenous fluids, bowel rest, parenteral nutrition, heparin prophylaxis and antibiotics), after 14 days, the condition of the patient deteriorated and surgery was necessary. Another atypical disease complication was the profuse episode of bleeding after 2 weeks of medical management. Treatment guidelines can be standardized depending on the depth of colonic mural ischaemia and its impact on vital function: surgical treatment is advocated for deep ischaemia with colonic necrosis and/or MOF, whereas observation and medical management are recommended for patients with superficial ischaemia without organ dysfunction [2]. Favier et al. described an endoscopic classification of IC, which included ischaemia limited to the mucosa with petechiae and small ulcerations with intervening healthy mucosa as stage I, ischaemia extending to the muscularis mucosa with large ulcerations as stage II and transmural ischaemia with necrosis of the muscularis and possible perforation as stage III [4]. Basically, in agreement with literature, patients with a confirmed IC diagnosis, without surgical abdomen and/or evidence of pneumoperitoneum, need surgery only if they have Favier stage 2 with MOF or Favier stage 3. A question could be why, in patients with ischaemia extending to the muscularis mucosa with large ulceration (Favier stage 2), we should wait for the appearance of sepsis, or MOF, before deciding on surgical treatment? We found, in the literature, how most patients with IC will clinically improve within 24 or 48 h. However, an indication for surgery is persistent symptoms beyond 2–3 weeks. Obviously, a septic patient, with MOF, or with perforation and/or peritonitis, has more risk of developing post-surgical complications than a patient who has surgery before the appearance of these conditions. A possible suggestion could be to decide on surgical management in all patients with Favier stage 2 in which there is no evidence of clinical improvement after 48–72 h. In our patient, we had a clinical improvement 6 days following admission but another worsening during the second week; this can suggest that the role of endoscopy and the grade of disease based on Favier classification could be a better prognostic index than a slow improvement in the clinical condition. Moreover, colonoscopy performed on table showed the extent of disease and allowed the right edges of resection to be identified. This is our conclusion after a comparison of our experience and a review of literature. However, we cannot reach a definitive conclusion without a sufficient sample of patients, but we would like to suggest a new possible strategy in the management of mild and severe IC, with the hope that it could be helpful for other studies in the future.
